# Resilience in childhood vaccination: analysing delivery system responses to shocks in Lebanon

**DOI:** 10.1136/bmjgh-2023-012399

**Published:** 2023-11-06

**Authors:** Sharif A Ismail, Andrada Tomoaia-Cotisel, Aya Noubani, Fouad M Fouad, Sadie Bell, Josephine Borghi, Karl Blanchet

**Affiliations:** 1 Department of Global Health and Development, London School of Hygiene and Tropical Medicine, London, UK; 2 RAND Corporation, Santa Monica, Los Angeles, USA; 3 Institute for Global Health and Development, Queen Margaret University, Musselburgh, UK; 4 Department of Epidemiology and Population Health, Faculty of Health Sciences, American University of Beirut, Beirut, Lebanon; 5 Geneva Center of Humanitarian Studies, University of Geneva, Geneva, Switzerland

**Keywords:** Immunisation, Health systems, Vaccines, Child health, Health systems evaluation

## Abstract

**Introduction:**

Despite rapidly growing academic and policy interest in health system resilience, the empirical literature on this topic remains small and focused on macrolevel effects arising from single shocks. To better understand health system responses to multiple shocks, we conducted an in-depth case study using qualitative system dynamics. We focused on routine childhood vaccination delivery in Lebanon in the context of at least three shocks overlapping to varying degrees in space and time: large-scale refugee arrivals from neighbouring Syria; COVID-19; and an economic crisis.

**Methods:**

Semistructured interviews were performed with 38 stakeholders working at different levels in the system. Interview transcripts were analysed using purposive text analysis to generate individual stakeholder causal loop diagrams (CLDs) mapping out relationships between system variables contributing to changes in coverage for routine antigens over time. These were then combined using a stepwise process to produce an aggregated CLD. The aggregated CLD was validated using a reserve set of interview transcripts.

**Results:**

Various system responses to shocks were identified, including demand promotion measures such as scaling-up community engagement activities and policy changes to reduce the cost of vaccination to service users, and supply side responses including donor funding mobilisation, diversification of service delivery models and cold chain strengthening. Some systemic changes were introduced—particularly in response to refugee arrivals—including task-shifting to nurse-led vaccine administration. Potentially transformative change was seen in the integration of private sector clinics to support vaccination delivery and depended on both demand side and supply side changes. Some resilience-promoting measures introduced following earlier shocks paradoxically increased vulnerability to later ones.

**Conclusion:**

Flexibility in financing and human resource allocation appear key for system resilience regardless of the shock. System dynamics offers a promising method for ex ante modelling of ostensibly resilience-strengthening interventions under different shock scenarios, to identify—and safeguard against—unintended consequences.

**WHAT IS ALREADY KNOWN ON THIS TOPIC**

- Research and policy interest in understanding dynamics influencing health system resilience is strong, but existing studies have focused primarily at macrolevel and on analyses of single shocks.

**WHAT THIS STUDY ADDS**

- This study presents the first analysis of vaccination delivery system responses to multiple, overlapping shocks in a humanitarian setting, using system dynamics.
- We show how shocks in Lebanon spurred potentially transformative change through task-shifting to support vaccination, but that early adaptive changes (eg, in the cold chain) may have undermined system resilience to later shocks.

**HOW THIS STUDY MIGHT AFFECT RESEARCH, PRACTICE OR POLICY**

- System dynamics offers a promising avenue for ex ante modelling of resilience-promoting measures, to identify potential unintended consequences.
- Rather than single interventions, meaningful promotion of long-term health system resilience likely requires packages of measures reflecting local contextual factors and varying in composition according to changing on-the-ground conditions.

## Introduction

Academic and policy interest in health system resilience has grown exponentially in recent years but the literature on this topic is emergent and empirical analyses of resilience are few.^1^ There is also uncertainty over how best to strengthen resilience, although it is acknowledged that doing so must extend beyond the more limited aims of health system support or health system strengthening.^2–4^

Understanding of resilience is particularly limited in humanitarian settings, where challenges to health system functionality can be acute, and resources to support meaningful responses to shocks may be more limited, unpredictable and dispersed than elsewhere. Delivery of essential services such as vaccination is challenging and liable to disruption.^5–7^ There is consistent evidence that childhood vaccination coverage for key antigens is among the lowest in the world for countries affected by conflict or other forms of humanitarian crises^8 9^ and key health outcomes often fall well below those observed in stable settings.^10–12^ Finally, existing guidance typically focuses on acute-phase response,^13 14^ rather than longer-term system resilience promotion.

In this study, we apply a resilience definition, distinguishing capacities to absorb, adapt or even transform in response to shocks, while maintaining system structures and continuing to deliver essential services.^15^ Absorption is a process in which no structural change occurs: the shock is simply accommodated using existing system structures and pathways.^15^ Adaptation can involve circumscribed structural or pathway changes. Transformative change, by contrast, involves harnessing learning to fundamentally alter system structure and strengthen it for the future.^16 17^

Although each of these aspects of resilience describes a process of change, most work in health systems research has adopted a static view focused on discrete resilience capacities (such as networking between system actors, the presence of multiple, alternate service pathways etc).^17–19^ A small number of studies have mapped out system vulnerabilities and mitigation measures in humanitarian settings.^20 21^ Finally, empirical explorations of transformation in health are very few.^1^ What system transformation means in practice continues to be a source of debate in other research disciplines.^22^

We used qualitative system dynamics to understand responses to shocks because this method focuses on aggregate-level, system behaviours contributing to outcome trends, and in particular, the role of feedbacks in explaining why systems behave as they do.^23–25^ The aim of our analysis was to identify vaccination delivery system responses and mitigation strategies to sequential shocks in Lebanon. We also considered the effects of interventions to support responses to earlier shocks on system resilience to later ones. The purpose for doing so was to identify points and pathways in the system through which responses were implemented and to consider what was learnt from efforts to strengthen resilience over time. Our primary focus was on supply side (service delivery) behaviours.

### Study setting

Lebanon is a small Middle Eastern country with an estimated population of just over 6 million in 2021.^26^ The health sector is fragmented with a dominant role for private providers in provision of both acute and preventive care.^27–31^ Principal vaccination access points include private clinics and dispensaries (pharmacies), charitably-supported facilities, the Ministry of Public Health’s (MoPH) primary healthcare centre (PHC) network and Social Development Centres under the auspices of the Ministry of Social Affairs. Implementing partner organisations (national and international NGOs) has historically played an important role in vaccination delivery through technical, material and financial support.

We focused on vaccination delivery system responses to four shocks (see timeline in online supplemental appendix 1), the first of which was refugee displacement from neighbouring Syria. As of 31 December 2022, there were some 815 000 Syrian refugees residing in Lebanon, down from a peak of just under 1.2 million in April 2015, but most displacement occurred from mid-2014 to early 2015. Second, from late 2019 onwards, Lebanon was affected by a compound shock, adding effects arising from COVID-19 and an economic crisis to long-term strains arising from population displacement. Finally, a large blast in the capital Beirut in August 2020 destroyed the national vaccine storage warehouse. For vaccination delivery specifically, data on administrative coverage for key antigens indicate large declines in national coverage broadly coinciding with the shocks described above.^32–35^

10.1136/bmjgh-2023-012399.supp1 Supplementary data

## Methods

This was a retrospective, qualitative system dynamics study drawing on analysis of semistructured interviews conducted in Lebanon—the case study setting for the work. System resilience was assessed in terms of population vaccination coverage for measles over time (ie, first and second doses of measles-containing vaccine or MCV1 and MCV2).

### Approach to primary data collection

#### Participant recruitment

Interviewees were sampled purposively from stakeholders working at national, regional and local level in Lebanon, including government (the MoPH), donors and agencies supporting the humanitarian response work in-country, implementing partners and service managers and practitioners involved in front-line primary care. Regional and facility-level data collection was designed to better understand dynamics affecting service delivery in two governorates in Lebanon, Beirut and Akkar, chosen to reflect the diversity of service delivery challenges in an urban (Beirut) and rural (Akkar) setting, respectively, and historical variations in health service access (much lower in Akkar than in Beirut). A substantial majority of participants (89%) were female. Table 1 provides a breakdown of interviews conducted.

**Table 1:** Breakdown of interviews conducted, by stakeholder group and timing, and mode of use in causal loop diagram development

Stakeholder group	Subcategory	Wave 1 (February–March 2020)	Wave 2 (July 2021–January 2022)	Analysis set	Validation set
National	Government	2	1	16	2
	Donors	0	4		
	Agencies	5	5		
	Private sector	0	1		
Regional bodies and implementing partner organisations		1	12	11	2
Local—facility level	Akkar	0	5	6	1
	Beirut	0	2		
Wave total		8	30		
Overall total		38		33	5

#### Interview design and conduct

In total, 38 semistructured interviews were carried out in 2 waves (February–March 2020 and July 2021–January 2022). Of these, eight were in-person and the remainder were carried out remotely via Zoom due to COVID-19 restrictions. Interviews gathered information on participant roles, generic structures supporting vaccination delivery, and system behaviours and policy responses identified as linked to the various shocks. Interviews were recorded and transcribed into MS Word. Most were performed in English by the lead author (SI); the small number of interviews performed in Arabic (all at facility level) were jointly conducted by SI and AN, transcribed, translated into English and then analysed. Interviews were then separated into two sets: one (n=33) for analysis and a reserve set (n=5) for validation.

#### Generation of the causal loop diagram (CLD)

The CLD was generated in three consecutive steps. In step 1, a CLD was developed for each individual interviewee to represent their mental model of shock effects on childhood vaccination delivery in Lebanon, and the range of system responses. Transcripts were coded using purposive text analysis to identify causal language.^36 37^ Coded segments were transferred into a predesigned MS Excel template^37^ and marked to identify variables and their causal relationships according to best practice in CLD diagramming conventions (see online supplemental appendix 2).^23 25^ These relationships were visually mapped using Vensim, a system dynamics diagramming and simulation modelling software tool.^38^ Finally, a preliminary pruning step was applied for each individual CLD in which only delays (where a material or information delay between two variables in a link was identified) and feedbacks (a circular link or set of links between variables) were retained in accordance with an approach outlined by Yearworth and White.^39^

In step 2, individual CLDs were combined using a stepwise process. First, diagrams were grouped according to the stakeholder set from which they originated (ie, national, regional and implementing partners and facility level) and then ordered in terms of their complexity.^40^ This was determined with reference to the number of (1) feedback loops, (2) delays, (3) links and (4) variables in each CLD, in that order. The most complex CLD in each set was used as the ‘anchor’ with which others were then combined sequentially, by adding variables, links, delays or loops identified as missing from the starting diagram. Another, pruning step was then carried out, retaining only delays and feedback loops with three or more causal links.^39^ This pruning step was used to help simplify the visualisation of causal chains within the aggregated CLD, using a standard process.

In step 3, the combined CLD was validated by comparing it to five individual CLDs generated from interview transcripts from the validation set, using RIQ analysis as above.^37^ Saturation in CLD development and validation was captured by tracking the number of additional variables, links and feedback loops introduced with each additional combination step^40^—results are reported in online supplemental appendix 3.

#### Analysis of the CLD

Vaccination delivery system responses to shocks shown in the CLD were classified using the absorptive–adaptive–transformative approach outlined in the introduction. Categorisation was based on assessment of the extent to which new system structures were introduced (transformation), new resources mobilised or existing structures modified (adaptation) as responses to the vulnerabilities identified elsewhere, with reference to the CLD.

## Results

In the sections that follow, we map some of the key pathways of system response to these shocks. A detailed list of system responses is given in online supplemental appendix 5, classified according to the shock following which they were introduced, and the health system building block targeted.

### System responses to refugee arrivals

#### Absorptive and adaptive responses

Immediate responses included macrolevel, mesolevel and microlevel mobilisation to address what was perceived as a high risk of vaccine-preventable disease (VPD) outbreaks (especially polio and measles) in the context of population movement. At the macrolevel, adaptive measures included national vaccination campaigns, launched in 2013 and 2014 with donor funding support. An early policy change concerned the relaxation of rules around access to care for refugees, so that displaced Syrians could access vaccination through publicly supported facilities at nominal cost (capped at LE3000, equivalent to around US$2 in 2018 prices). The motivation for this change was to stimulate demand by addressing cost barriers. However, hidden fees (eg, for consultations) continued to create barriers to access for refugees and other vulnerable populations:

The product is free, however, it is very important to know that a major challenge that is facing the accessibility of refugees and vulnerable populations to free vaccination is sometimes the hidden fees. [LFS01, agency representative]

Two additional policy changes were introduced to improve access to vaccination. First, vaccination points were established linked to border crossing sites and refugee registration centres to allow for administration of key vaccines (oral polio vaccine and measles) to displaced Syrians either as they arrived in Lebanon or as they registered for access to services through the United Nations High Commissioner for Refugees (UNHCR). Second, the MoPH-supported PHC network was progressively expanded. Facilities in the network received no direct funding from the MoPH, but in exchange for ministry accreditation received vaccine doses free of charge (procured via UNICEF) and capacity-building support. A principal condition of membership of the network was to administer vaccines according to the national schedule at the fee rates set out nationally (see above).

**Figure 1:**
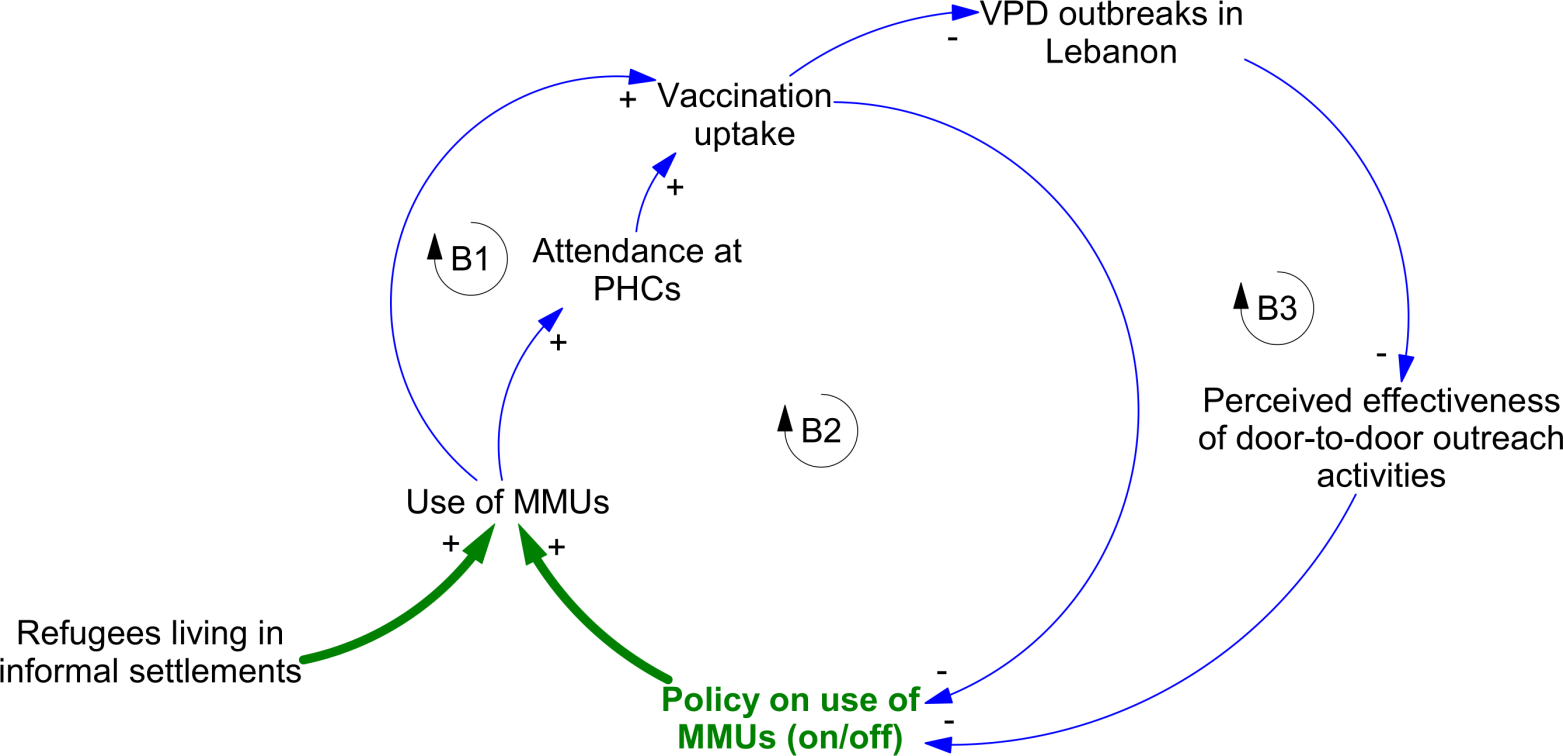
Dynamics influencing the use of mobile medical units (MMUs) in Lebanon over time. Active system response pathways and policies are highlighted in green. Regular system pathways are shown in blue. Arrowheads indicate the direction of causal relationships between variables. Polarities (‘+’ or ‘−’) indicate the nature of the causal link between variables—that is, whether an increase in the first variable leads to an increase or decrease in the second. Feedbacks labelled ‘B’ etc are balancing loops. PHC, primary healthcare centre; VPD, vaccine preventable disease.

Mesolevel changes—again predominantly adaptive—also occurred. A key initial response from implementing partners was intensified the use of mobile medical units (MMUs) (figure 1). These had historically been used for outreach to marginalised and remote populations in Lebanon. MMUs were now used to enable access for displaced Syrians who tended to settle in rural areas in the North and East of Lebanon where access to fixed-site clinics was more limited (loop B1), but also to facilitate referral in to PHCs where those facilities were available locally (loop B2).

The use of MMUs was progressively scaled back from 2017, however, as part of a strategy of encouraging refugees and host communities into fixed-site clinics instead (loop B2). Interviewees highlighted concerns regarding the effectiveness of the MMU model including perceived challenges regarding quality of care and a supposed dampening effect on motivation of service users to attend fixed sites:

At the beginning of the Syrian influx, there was vaccination at the borders, and there were a lot of campaigns and having mobile units going to the places of…Syrian groups and providing them there with vaccines. So they thought…we can stay home and the medical teams will come to us and provide vaccines and we don’t need to go, so they were a little bit passive. [LFS12]

Following a new measles outbreak in 2018–2019, doubts also emerged regarding the cost-effectiveness of this delivery approach (loop B3).

Over time, community engagement activities were stepped up to strengthen demand by (1) reinforcing the importance of vaccination as a preventive measure, (2) offering opportunities to cross-check vaccination records to make sure children were up to date with the national vaccination schedule (3) and strengthening household-level knowledge in areas likely to promote a decision to vaccinate—including information on the national schedule, on the actual cost of vaccination to refugees through the PHC network, and the location of the nearest PHC(s). These activities depended on stepped up recruitment of community engagement workers and increases in implementing partner funding—both of which depended on donor funding that took time to mobilise.

Microlevel (within facilities) changes revolved principally around changes to the role of nurses in vaccine administration, as set out in the next section. However, interviewees also highlighted the importance of progressive improvements in workforce skills at facility level as clinic staff became more accustomed to managing increased workload and to better attuned to needs among refugee populations.

### Transformative change: task-shifting for vaccination delivery

**Figure 2:**
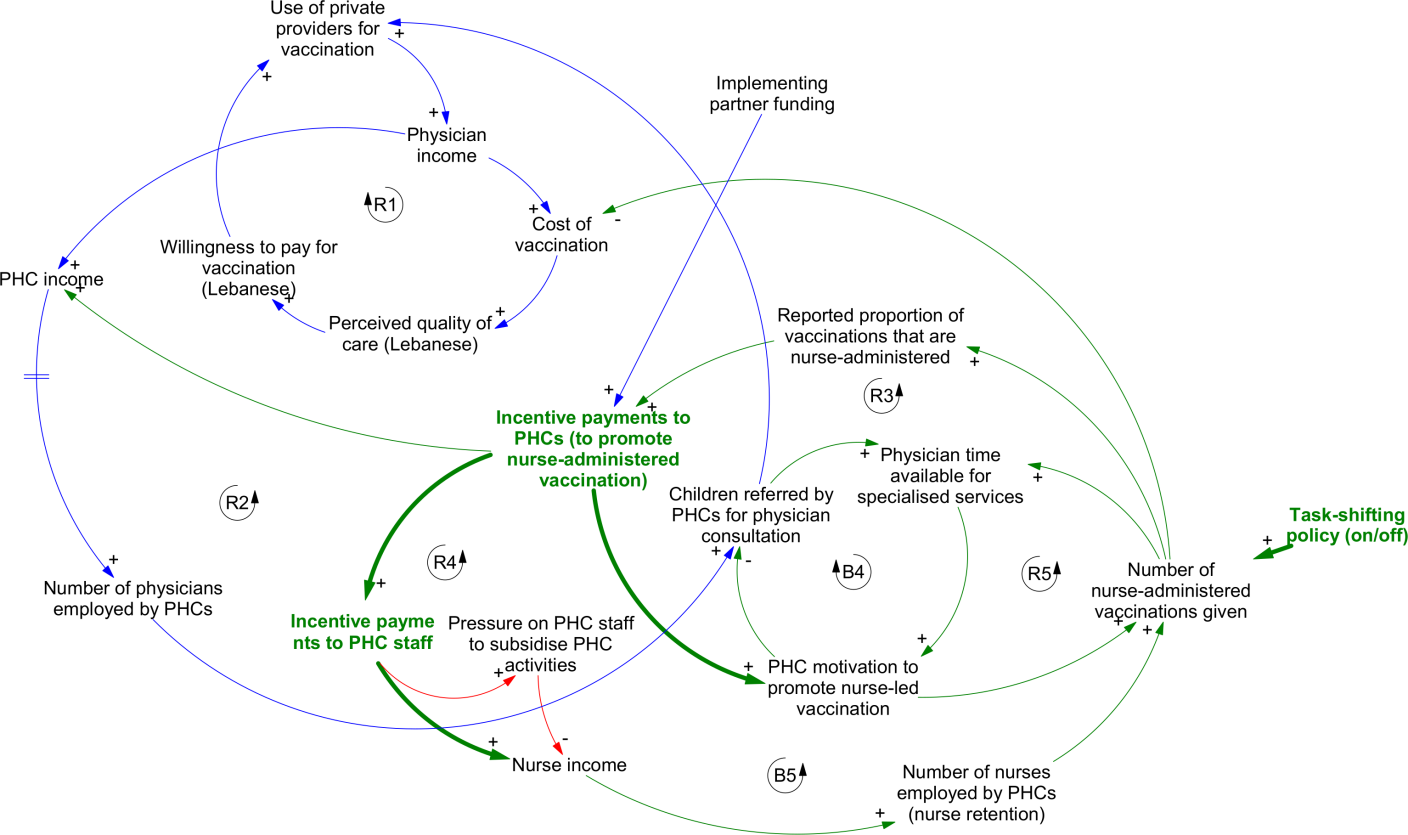
Dynamics linked to the introduction of task-shifting for vaccination delivery in primary healthcare centres (PHCs). Loops in blue show pathways present prior to the refugee crisis; those in green indicate new structures that emerged as part of the system response. Bold green lines and green variables indicate the principal points of intervention; light green lines indicate downstream effects. Red lines indicate new areas of risk that emerged following the introduction of the interventions. Arrowheads indicate the direction of causal relationships between variables. Polarities (‘+’ or ‘−’) indicate the nature of the causal link between variables—that is, whether an increase in the first variable leads to an increase or decrease in the second. Feedbacks labelled ‘B’ are balancing loops; those labelled ‘R’ are reinforcing.

Task-shifting to nurse-led vaccine administration was a key response to refugee arrivals (figure 2), combining a macrolevel policy change around health workforce regulation and supporting mesolevel changes to incentivise a change in delivery behaviour. Interviewees identified two motivations for this change: (1) a desire to reduce the cost of vaccination especially for refugee populations but also for host communities because physicians tended to charge over the odds for vaccine administration and (2) better workload management given rising demand in a system that previously had relied almost entirely on physician-administered vaccination.

Progress in implementing task-shifting was, however, limited by the availability of financial material resources to support it initially. This ensured that previously dominant loops favouring physician-led administration (loops R1 and R2) continued to drive most vaccination delivery. Physician administration of vaccines was an important source of revenue for both clinicians, and for PHCs that hosted them. On one hand, a long-standing preference among host communities for private medical care (including for vaccination) ensured steady revenues for physicians from private vaccination delivery. On the other hand, PHC managers who referred children to physicians privately for vaccine administration could expect to receive a cut from the resulting consultation fees.

The introduction of incentive payments to PHCs (via implementing partners) helped to ensure that task-shifting at facility level was finally implemented. It did so by (1) providing additional income to PHCs to offset losses as fewer children were referred to private physicians, (2) increasing PHC motivation to promote nurse-led administration to patients in the wider context of scepticism especially from host communities (loop R3), (3) freeing up physician time for more lucrative specialised activities (loop B4) and (4) indirectly supporting nurse pay and thereby improving their retention at facility level. Implementing partners tested various modes of incentivisation, including payments directly to nurses, but these were found to increase the risk of pressure on staff to subsidise other PHC activities (loop B5), so they were phased out in favour of payments to PHC managers to then distribute among nursing staff (loop R4).

### System responses to compound crisis

#### Absorptive and adaptive responses

Interviewees described a range of responses to COVID-19, many of them operating at microlevel. In the near term, there were absorptive responses to the perceived risk of contracting infection in facilities, influenced by perceived inability to socially distance and by health worker concerns about the probability of exposure in a context of rising caseloads nationally. Ad hoc measures included local clinic cancellations and reductions in staffing levels to reduce the potential for health worker exposure. However, these reduced access opportunities for patients in the short-term, contributing to declines in vaccination uptake. At national level, interviewees highlighted the concentration of resources on COVID-19 surveillance at the expense of monitoring for other VPDs, increasing the risk of delayed outbreak detection.

Responses elsewhere were adaptive and primarily focused on reinforcing supply and demand through improved risk management across the system, although lead times to implementation were longer. Stocks of personal protective equipment were mobilised and staff trained in infection prevention and control introduced to reduce health worker perceived risk of contracting infection in health facilities. On the demand side, community engagement activities continued but were shifted online or via tools such as WhatsApp, although quality of engagement with service users was perceived as poorer as a result of this. Messaging focused on reinforcing the importance of vaccination as a preventive service, and emphasising that clinics remained open despite COVID-19-related movement restrictions.

**Figure 3:**
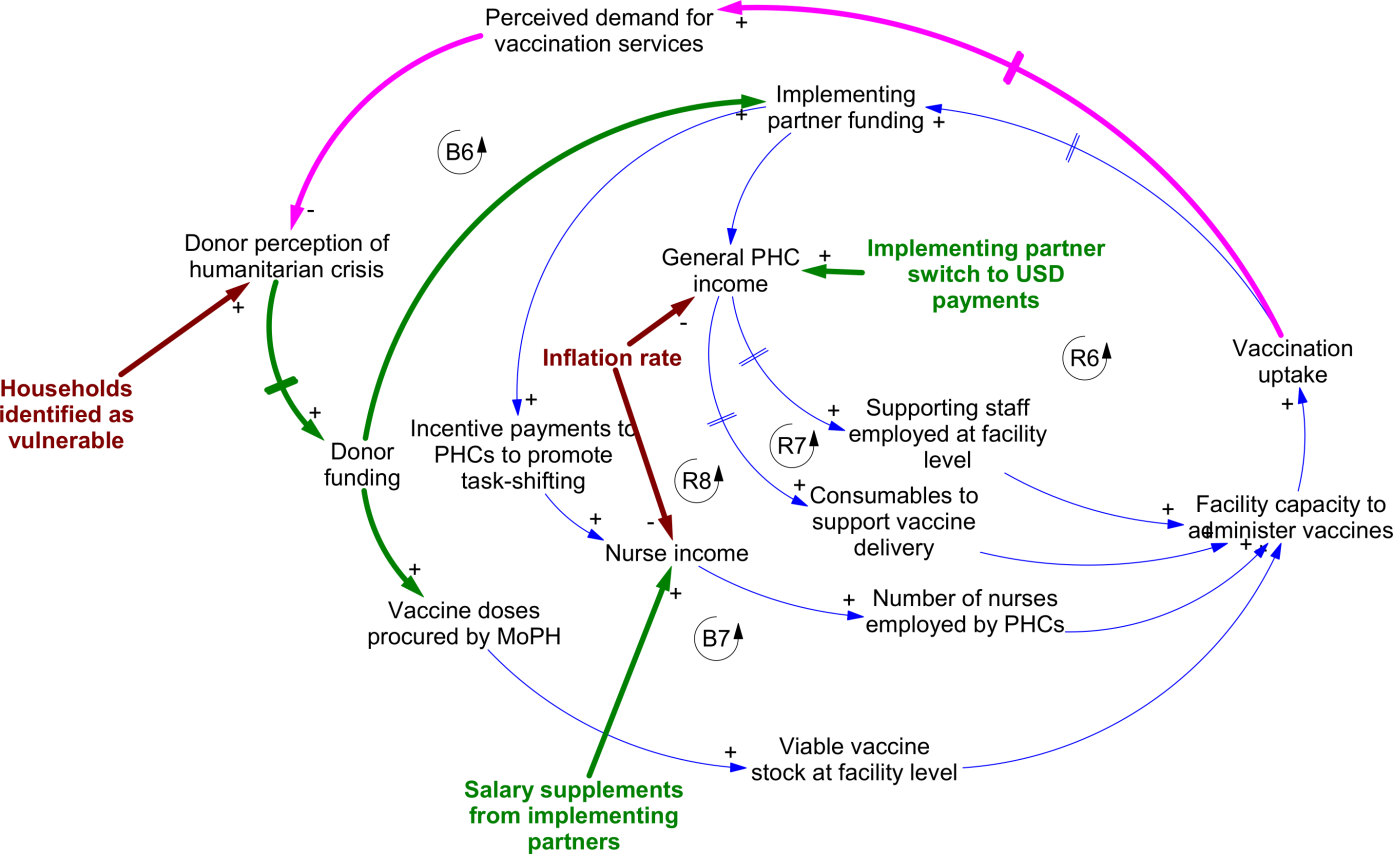
Dynamics linking changing perception of need to mobilisation of donor funding, and downstream adaptive responses within the system. Lines in bold pink correspond to pathways of impact for COVID-19; lines and text in bold brown correspond to those for the economic crisis. Intervention points are highlighted in bold green text; key response pathways feeding into established system loops are shown using bold green lines. Arrowheads indicate the direction of causal relationships between variables. Polarities (‘+’ or ‘−’) indicate the nature of the causal link between variables—that is, whether an increase in the first variable leads to an increase or decrease in the second. Feedbacks labelled ‘B’ are balancing loops; those labelled ‘R’ are reinforcing. MoPH, Ministry of Public Health; PHC, primary healthcare centre.

Responses to the economic crisis were similar in nature but generally targeted different pathways within the system (figure 3). Adaptive measures focused on maintaining facility capacity to deliver vaccination through additional financial support as currency inflation worsened. These included switching the currency of implementing partner payments from Lebanese currency to US dollars, to offset declining PHC income due to inflation. The goal was to maintain PHC income and therefore capacity to deliver (loops R6 and R7). Some implementing partners also provided direct salary supplements to PHC staff to improve retention (loop R8). Financial support measures did not, during this period, extend to incentives for service users.

Mobilisation of donor funding was a key adaptive response to the compound crisis. This was triggered by (1) changes in the COVID-19 caseload, (2) a collapse in demand for childhood vaccination observed especially during the first COVID-19 lockdown in Lebanon (loop B6), (3) an emergency appeal following the Beirut blast and (4) needs assessments identifying rapid growth in the proportion of households (refugee and host communities alike) identified as vulnerable (loop B6 in figure 3). Emergency funding following the blast was mostly focused on greater Beirut, where the damage was greatest. However, a shift in donor perception—to recognising the economic crisis as a *humanitarian* one—contributed to strengthening resource mobilisation to PHCs nationwide via implementing partners. It also helped support vaccine procurement to maintain delivery through the PHC network, and—as the following section outlines—recruitment of private sector capacity to support childhood vaccination delivery. However, interviewees highlighted the length of time it took both for declines in vaccination uptake to be identified, and for the implications of the economic crisis for health needs to be recognised. This contributed to delays in funding mobilisation.

### Transformative change: integrating private sector delivery capacity

**Figure 4:**
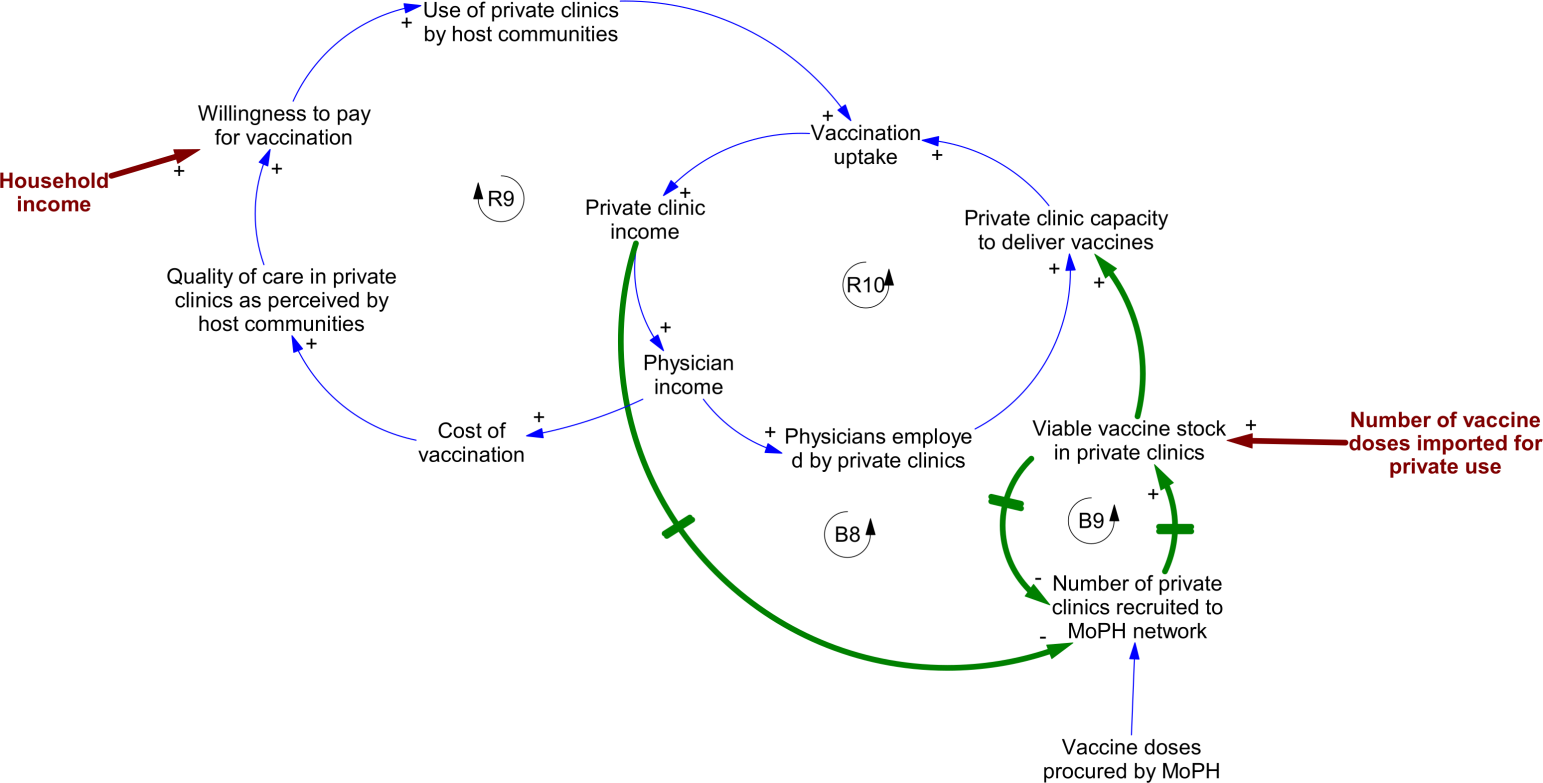
Dynamics linked to private clinic integration into the publicly supported system for delivery of routine antigens at nominal cost to patients. Points of shock interaction are denoted by brown text and arrows; loops in blue show pathways present prior to the three shocks; lines in bold green indicate new pathways introduced through policy interventions from 2015 onwards. Arrowheads indicate the direction of causal relationships between variables. Polarities (‘+’ or ‘−’) indicate the nature of the causal link between variables—that is, whether an increase in the first variable leads to an increase or decrease in the second. Feedbacks labelled ‘B’ are balancing loops; those labelled ‘R’ are reinforcing. MoPH, Ministry of Public Health.

Interviewees described opportunities created by the shocks—in particular the integration of private sector delivery capacity to support childhood vaccination delivery at nominal cost (figure 4). Although the MoPH had introduced a policy to promote private sector engagement in vaccination delivery in 2015, this did not attract meaningful engagement until the economic crisis took hold. Interviewees ascribed this partly to host communities’ continuing belief that cost and quality of care were linked, and resulting tendency to take their children to private clinics for vaccination (loop R9). The lucrative nature of private vaccine administration for physicians also contributed (loop R10). Because of this, incentives for private clinics to participate in the scheme were weak (loop B8).

However, vaccination uptake through private clinics collapsed in 2021/2022. This was the combined result of falling household incomes, and the increasing difficulty clinics experienced in sourcing vaccine doses through the open market due to import restrictions. Declining income as a result of this encouraged increasing private clinic participation in the MoPH’s scheme. In exchange for free vaccine doses (thereby increasing the stock of viable doses in clinics—loop B9), private clinics were required to drop the cost of vaccination to levels equivalent to those in PHCs and to report vaccination uptake data to the MoPH.

### Pathways of interaction across shock responses

**Figure 5:**
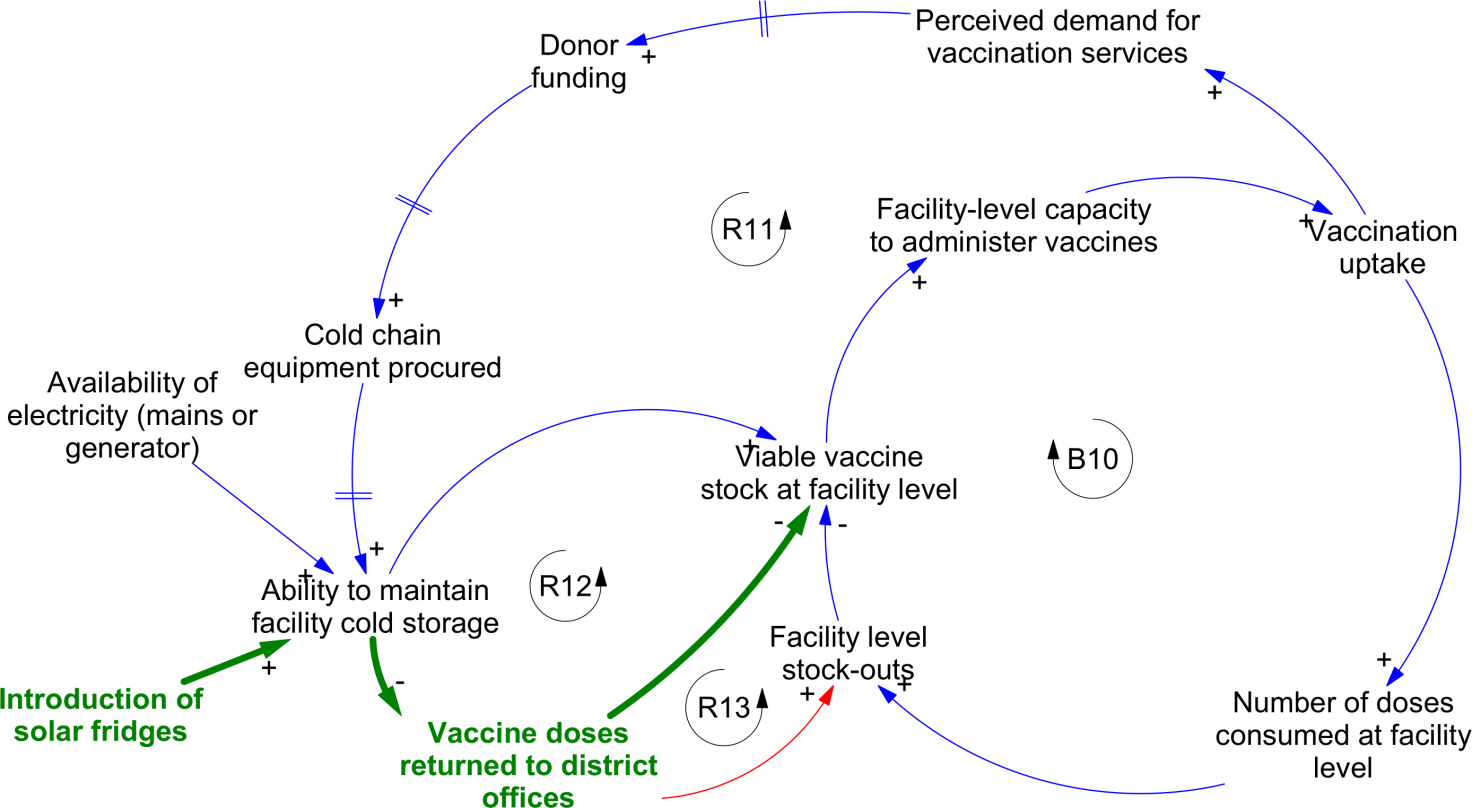
Interactions between early responses and later shocks contributing to vulnerabilities in the cold chain. Dark green text and lines indicate interventions introduced to support system responses, and red lines indicate new areas of risk linked to those interventions. Arrowheads indicate the direction of causal relationships between variables. Polarities (‘+’ or ‘−’) indicate the nature of the causal link between variables—that is, whether an increase in the first variable leads to an increase or decrease in the second. Feedbacks labelled ‘B’ are balancing loops; those labelled ‘R’ are reinforcing.

Interviewees highlighted three areas in which responses to earlier shocks contributed to potentiating the impact of later ones. The first was cold chain integrity (figure 5), where a key adaptive response to gradually rising demand following refugee arrivals, and ongoing insecurity of mains electricity supplies, was the introduction of solar fridges. This improved cold storage capacity in PHCs in the near term and enhanced facility capacity to deliver vaccinations (loop R11), but import restrictions linked to the economic crisis contributed to difficulties in sourcing spare parts for these fridges. Combined with the increasing unreliability of mains electricity supplies and the scarcity of generator fuel, PHC staff found it harder to guarantee cold chain integrity. Locally, doses were increasingly returned to district-level storage facilities, where cold storage was more reliable (loop R12), but this increased the risk of facility-level stockouts and reduced the capacity of facilities to respond to fluctuating local demand (loop R13).

Challenges were also noted for electronic record systems, originally introduced as a mechanism for improving service efficiency and record completeness following refugee arrivals from Syria. As electricity supplies became more unreliable, facilities sometimes had to revert to paper-and-pen methods for which later online reconciliation would be needed to ensure records were complete:

Because now we are working on an electronic system…sometimes to search the name of the child or their parents and to check all the vaccines received by this child at the centre or elsewhere previously, it’s difficult due to the cut or interruption of internet. [LFS11, government official]

A third point of interaction concerned donor and agency policy regarding financial support for salaries. In 2016 and 2017, a decision was taken to provide direct salary support for posts in the MoPH to support the vaccination programme (among others), boosting central oversight and programme support capacity. However, this policy was later reversed contributing—alongside a general government hiring freeze—to severe staffing shortages:

[Agency X] alone supported us with like 100 staff on [district] and central level for two years, 2016, 2017. And they stopped…so now we are in real, real shortage of staff at central and at peripheral level…because all the donors, they don’t want to pay for staffing, they only want to pay for activities, and we cannot do activities without staffing. [LFS03, government official]

On the other hand, workforce attrition among nursing staff was lower than for physicians as economic conditions worsened. In this sense, task-shifting in vaccination delivery was important in strengthening resilience to a series of shocks—refugee arrivals in the first instance, and then the economic crisis.

## Discussion

To our knowledge, this is the first study to apply system dynamics to investigate shock responses to support vaccination delivery system resilience in a humanitarian setting, and the first to address multiple, overlapping shocks. Responses occurred at multiple system levels, and almost all were adaptive—via mobilisation of additional financial, human or other resources. We identified two potentially transformative changes, the first being a policy change to bring some private clinics under the MoPH’s fold for lower-cost access to vaccines. This policy was introduced in 2015 but did not result in significant behavioural change until demand collapsed in the private sector in 2021/2022, helping to push host communities towards PHCs for vaccination and other health services. The second was the implementation of task-shifting to nurse-led vaccine administration in PHCs. Although neither of these resulted in large-scale goal reorientation across the system, both introduced new mesolevel and microlevel system pathways promoting vaccination delivery that had not previously existed.

System responses were sometimes maladaptive for vaccination uptake. Many early responses to COVID-19 (eg, reduced clinic working hours, reducing clinic staffing levels) helped preserve workforce well-being in the short-term and contributed to reducing transmission of infection, but also reduced service access opportunities for patients. The greater focus on absorptive responses to COVID-19 and particularly the economic crisis by comparison with earlier changes following refugee arrivals from Syria (see online supplemental appendix 5) emphasised the extent to which the limits of system capacity to accommodate disruptions had been reached. Interviewees repeatedly highlighted the dependence of the system on donor and implementing partner resources to continue functioning.

Our findings also show points of interaction between system responses, and ways in which changes implemented to address effects arising from earlier shocks influenced vulnerability to later ones over the long term. The introduction of imported solar fridges into the cold chain, for example, made an important contribution to strengthening cold storage capacity at facility-level following the refugee arrivals, but growing difficulties obtaining spare parts as import restrictions intensified in 2021/2022 increased cold chain vulnerability at a time when mains and generator electricity supplies were also less and less reliable. By contrast, the use of task-shifting reinforced long-term system resilience by expanding delivery capacity but also because nurses proved less likely than their physician colleagues to leave their posts or emigrate despite deteriorating economic conditions. These challenges may reflect the primarily technical, absorptive and adaptive emphasis of interventions introduced in response to earlier shocks in Lebanon, as opposed to transformative changes likely to reinforce system resilience to a range of shocks in the long term and especially ones of the scale and scope of the economic crisis. The need for attention to long-term time horizons in formulating appropriate approaches to supporting system resilience is well recognised in the system dynamics literature and should be a focus of response and recovery work in Lebanon and other humanitarian settings.^41^

Findings also support those identified in other empirical analyses of health system resilience in humanitarian settings. Our work underscores the need for flexible approaches to human resource management,^20 21^ and the importance of decentralisation for adaptive responses in some areas.^20 21 42^ Financing decentralisation proved both a strength and a weakness in Lebanon. On one hand, interviewees highlighted the threat posed by donor fatigue and limited resilience within the system to cope with declines in external funding. On the other hand, the diversity of financing streams proved essential to maintaining vaccination delivery as economic conditions worsened in 2021/2022. Implementing partners stepped in to provide additional financial resources (sometimes in foreign currency) and technical support in a way that would have been impossible for public actors to do given a government hiring freeze and deepening fiscal crisis.^43^ Implementing partners could also mobilise funds to facilities quickly.

Findings also mirror those elsewhere regarding the importance of parallel service delivery pathways^21^: multiple service delivery modalities were used at various stages to bolster uptake, including MMUs, fixed site PHCs, border crossing sites, registration site clinics and national campaigns among others. The importance of timely information flows to support situation appraisal is also clear.^17^ Delays to recognition of critical changes in behaviour (eg, the time taken to identify changes in service demand and the time taken for donors to formally recognise unfolding crises) both imposed significant limits on vaccination delivery system responsiveness.

Limitations to this analysis include that a detailed evaluation of demand side responses, drawing in service user perspectives, was not attempted for reasons of practicality and representativeness across populations in Lebanon. Insights on community-based resilience strategies are therefore limited, although anecdotal evidence indicates these have been important during the economic crisis. Second, recall bias may have affected reporting of system responses to refugee arrivals, given the time that elapsed between peak cross-border movement (in 2014/2015) and this analysis. Measures taken to minimise this included recruiting participants with a spectrum of experience in the system (ranging from a few years to several decades) and inviting participants to focus only on areas where their recollections were strongest.

We highlight a number of policy implications from this analysis. Evidence in this study emphasises the extent to which long-term system resilience depends on delivery using a variety of service delivery models and underscores the importance of continual demand reinforcement through community engagement (for which multiple strategies were used in Lebanon). It also underscores the need for measures to address multisystemic risk. Household income proved to be a key determinant of demand and changes contributed to fundamental shifts in patterns of demand in Lebanon in 2019–2022.

On the supply side, our findings emphasise that reactive campaigns are likely to remain a mainstay of efforts to improve vaccination coverage in acute and protracted humanitarian settings, especially because population movement often does not immediately translate into increased demand for preventive services. Second, policy changes introduced at macrolevel need to be supported by appropriate, and suitably financed, cascaded actions to ensure on-the-ground implementation. This was evident in the initially slow progression of task-shifting to nurse administration, a policy that proved hard to implement in the face of powerful vested interests without direct incentivisation at mesolevels and microlevels. Finally, policies should be designed for the long term. Although the mutability of on-the-ground conditions in humanitarian settings makes this challenging, we identified examples where external actions amplified long term risks—for example, the decision to import solar fridges with difficult to access spare parts in an import-heavy economy, and a ‘boom-and-bust’ pattern of agency support for civil service salary financing.

## Conclusion

Flexibility in financing and human resource allocation was key in enabling the publicly supported childhood vaccination delivery system in Lebanon to continue functioning despite accumulating vulnerabilities in the face of overlapping shocks, as was structural transformation in discrete areas of the system. However, by early 2022, the wide-ranging effects of the economic crisis on both demand side and supply side dynamics appeared close to overwhelming the compensatory effects of response mechanisms and highlighted the growing dependence of the system on external support.

## Data Availability

No data are available. Due to the sensitive nature of the questions asked in this study and risk of identification, participants were assured raw data would remain confidential and would not be shared.
