# Supplementary material for: Resilience in childhood vaccination: analysing delivery system responses to shocks in Lebanon
Source: BMJ Glob Health. 2023 Nov 6;8(11):e012399. doi: 10.1136/bmjgh-2023-012399 (PMC10632819; doi:10.1136/bmjgh-2023-012399)
Supplement: Supplementary data [file bmjgh-2023-012399supp001.pdf]

Supplementary materials

Appendix 1: Timeline of key events

Wider contextual developments	***Year***	***Vaccination delivery system developments***
National accreditation programme for PHCs launched	2009	
Beginning of Syria Crisis	2011	
Beginning of large-scale cross-border population movement<br><br>March: inter-agency regional response plan to Syria Crisis initiated<br><br>Registered refugee numbers increase from circa 12,000 in Feb/March to 156,000 by end December	2012	Implementing partner NGOs begin vaccine delivery to displaced populations through MMUs, in partnership with MoPH
Cases of AFP reported in Syria; polio vaccination campaign launched<br><br>804,848 registered refugees by 30/12/2013	2013	Nationwide measles and mumps outbreak reported<br><br>National cluster survey for routine vaccination coverage carried out but does not include data on Syrians specifically
Dec: EU regional trust fund launched to support delivery of donor funds for projects in Lebanon and other countries, including for health<br><br>1,146,405 registered refugees by 31/12/2014	2014	April: MoPH issues circular requesting vaccination be administered free of charge at all PHCs<br><br>May: MoPH issues circular promoting cooperation with private providers including through provision of vaccine doses procured through the Ministry<br><br>Nationwide measles and mumps outbreak continues
Lebanese Government requests UNHCR stop registering new refugee arrivals in Lebanon<br><br>1,069,111 registered refugees by 31/12/2015	2015	Jan: LCRP launched – surge in donor commitments through the plan<br><br>May: MoPH issues circular reinforcing shift from physician-administered to nurse-administered routine vaccination in PHCs (without consultation fees)

Ismail SA, *et al. BMJ Glob Health* 2023; 8:e012399. doi: 10.1136/bmjgh-2023-012399

1,011,366 registered refugees by 31/12/2016	2016	National cluster survey for routine vaccination coverage carried, disaggregating Syrian and host community coverage rates for the first time
Cases of AFP reported in Syria; polio vaccination campaign launched<br><br>June: Lebanon Health Resilience Project (World Bank) launched, with module focusing on primary care service strengthening<br><br>997,552 registered refugees by 31/12/2017	2017	Donor policy shift from funding staff and overhead costs to prioritising programmes; funding for MoPH staff reduced<br><br>Revised National Immunisation Strategy and EPI multi-year plan of action issued
948,849 registered refugees by 31/12/2018	2018	Nationwide measles outbreak reported<br><br>PHENICs e-record system launched<br><br>Policy shift away from use of MMUs towards promotion of vaccination uptake for all populations through PHCs and other fixed sites
October: protest movement begins; Lebanese currency begins slide in value against US dollar<br><br>914,648 registered refugees by 31/12/2019	2019	National measles vaccination campaign launched with donor support<br><br>Mobile Epi Registry Application (MERA) e-record system launched
February: first COVID-19 cases reported in Lebanon<br><br>March-May: first COVID-19 lockdown<br><br>August: second COVID-19 lockdown; Beirut blast<br><br>865,531 registered refugees by 31/12/2020	2020	MoPH launches programme to contract private clinics to deliver routine vaccinations free of charge, in exchange for vaccine dose supplies<br><br>Large decline in reported vaccination uptake through PHCs<br><br>National vaccination storage facility transferred from Karantina to Rafiq Hariri University Hospital in Beirut to preserve cold chain following Beirut blast
November: government subsidies on the costs of regular medications lifted<br><br>840,929 registered refugees by 31/12/2021	2021	
825,081 registered refugees by 30/09/2022	2022	Expanded use of MMUs to meet local population demand for vaccination

Ismail SA, et al. BMJ Glob Health 2023; 8:e012399. doi: 10.1136/bmjgh-2023-012399

Appendix 2: Introduction to causal loop diagrams

Causal loop diagrams (CLDs) show cause and effect relationships between variables in a system. In a CLD, variables are linked by arrows which indicate both the direction of perceived effects, and – through the polarity (as indicated by a '+' or '-' sign) – the nature of the relationship. A positive polarity indicates a reinforcing relationship between variables so that as one increases, the linked variable does in turn. A negative polarity indicates an opposing effect, so that a rise in the value of the starting variable leads to a reduction in the resultant variable.

The use of hashed lines across an arrow linking two variables indicates a delay in movement from one to the next. These delays can broadly be of two types: (i) *information delays*, which describe the time taken to assemble and interpret relevant information that enables a system change to be perceived or acted upon; and (ii) *material delays*, which describe the time taken for movement of materials affecting downstream system behaviours. Supply chain and logistics problems are good examples of material delays; in the case of vaccination delivery, one might consider the delay between disbursement of vaccine doses from a central storage facility to their arrival at a health service provider for administration, and the range of potential factors contributing to this.

Figure 1 provides a simple illustration, showing the link between two variables used in this project: *PHC crowding*, and *Perceived ability to socially distance in PHCs*. In this example, as PHCs become more crowded, service users' confidence in being able to socially distance properly within those facilities reduces. There is also an information delay, in that it takes time for service users to become aware that local health facilities are crowded (unless they happen to be present in person).

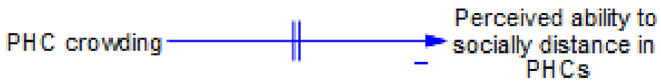

Figure 1. Illustration of a negative polarity relationship between two variables using standard CLD notation. This visualisation shows the link between primary healthcare facility crowding and service user-perceived ability to socially distance in those facilities (in the context of COVID-19 spread).

For more complex behaviours, variables may be linked together to form loops, in which feedback behaviour occurs. These can be *reinforcing* or *balancing*. In a reinforcing loop, a series of variables may be linked together by positive polarities, so that a positive feedback loop is created. These kinds of loops can lead to rapid growth or decline in the variables of interest over time. A balancing loop, by contrast, tends towards equilibrium, and is seen when the relationships between variables within a loop even each other out. Further detail on the

interpretation of CLDs can be found in a number of sources including Tomoaia-Cotisel *et al* (1), or for those with time and interest to pursue more detail, in Sterman (2) or Morecroft (3).

Figure 2 provides an illustrative example of a balancing feedback loop, based on the stem given in Figure 1. In this example, an increase in the perceived ability to socially distance in health facilities, reduces the perceived risk of contracting COVID-19, in turn promoting attendance at those facilities, with a resulting increase in vaccination uptake. However, there is a feedback effect because as attendance rises, so too does PHC crowding, which tends to reduce perceived ability to socially distance, ultimately discouraging further PHC attendance.

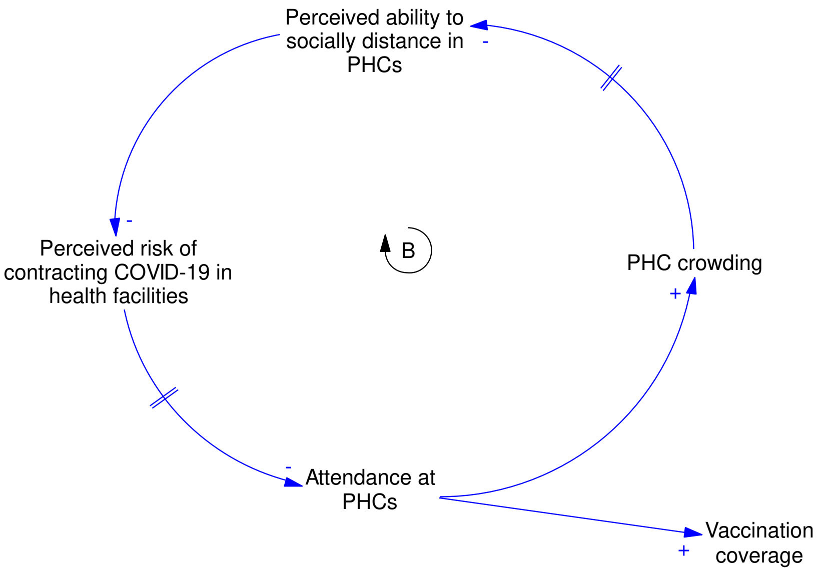

Figure 2. Example of a balancing feedback loop.

References

1. Tomoaia-Cotisel A, Kim H, Allen SD, Blanchet K. Causal loop diagrams: a tool for visualizing emergent system behaviour. In: de Savigny D, Blanchet K, Adam T (eds.) *Applied Systems Thinking for Health Systems Research*. London: McGraw Hill; 2017. p. 97–114.

2. Sterman JD. *Business Dynamics: Systems Thinking and Modeling for a Complex World*. McGraw-Hill Companies Inc; 2000.

3. Morecroft JDW. *Strategic modelling and business dynamics: a feedback systems approach*. Chichester: Wiley; 2007.

Ismail SA, *et al. BMJ Glob Health* 2023; 8:e012399. doi: 10.1136/bmjgh-2023-012399

Appendix 3: Tracking saturation through CLD combination and validation

In order to test the extent to which saturation had been reached in development of combined CLDs from different stakeholder groups, saturation plots were generated for both CLD combination (Figure 3), and subsequent validation of the final, aggregated CLD (Figure 4). These plots are intended to give a quantitative sense of the contribution of new manuscripts to elaborating the CLD, and measure, respectively, the number of new variables, links, delays and feedbacks introduced with each additional CLD. As CLD development proceeds to saturation, one would expect the number of new additions with each transcript to tend towards zero.

The plots overleaf for CLD combination give a visual indication of the extent to which saturation was reached across stakeholder groups. Clusters correspond to the stakeholder categories identified in the methods section of the main manuscript. The second set of figures visualises saturation in CLD validation using the validation set of interviews.

Ismail SA, *et al. BMJ Glob Health* 2023; 8:e012399. doi: 10.1136/bmjgh-2023-012399

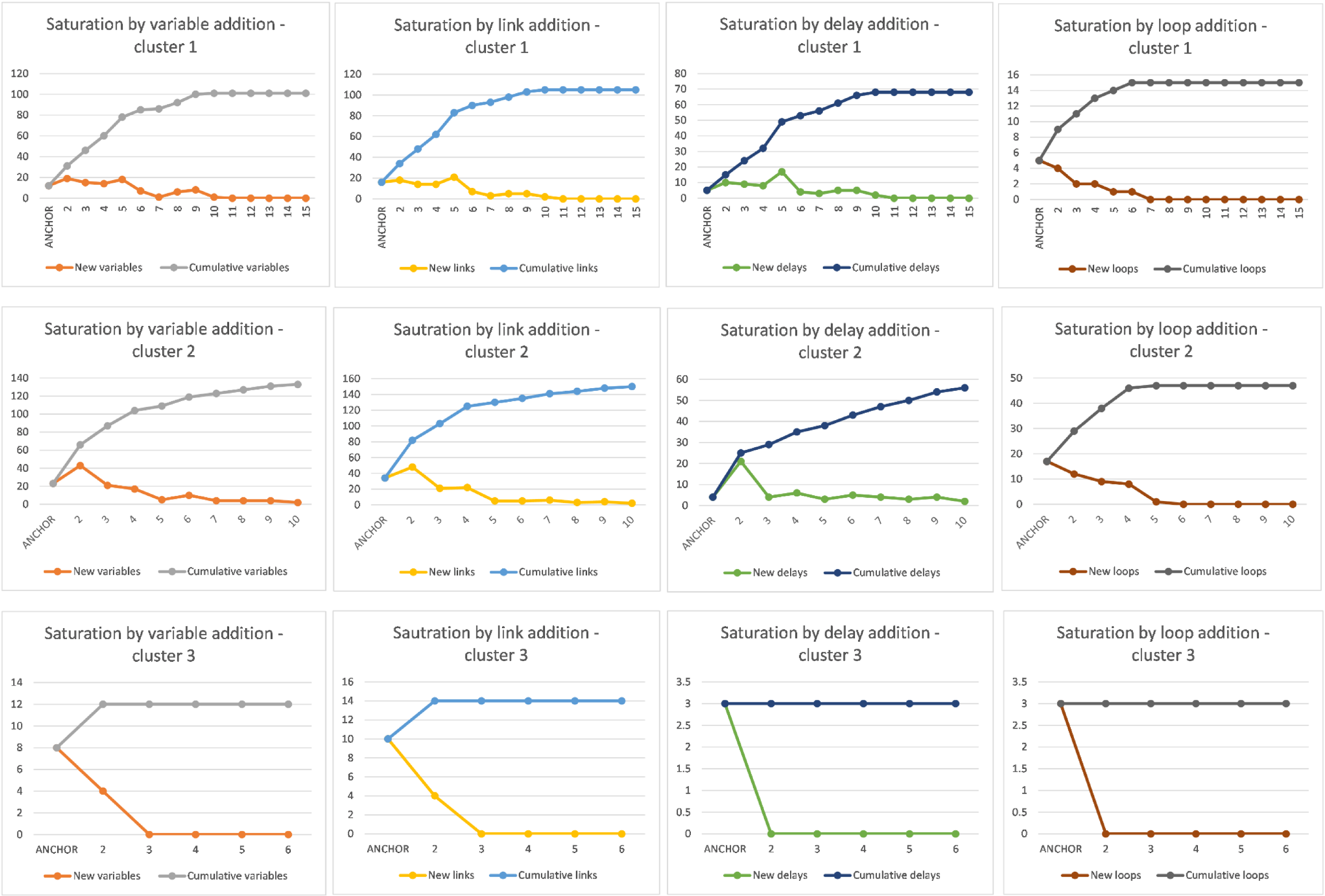

Figure 3. Saturation curves by variable addition, link addition, delay addition and feedback loop addition for each additional CLD added from the anchor CLD during the CLD combination phase.

Clusters correspond to the stakeholder category (where cluster 1 = national level stakeholders; cluster 2 = regional stakeholders and implementing partners; and cluster 3 = facility-level interviewees).

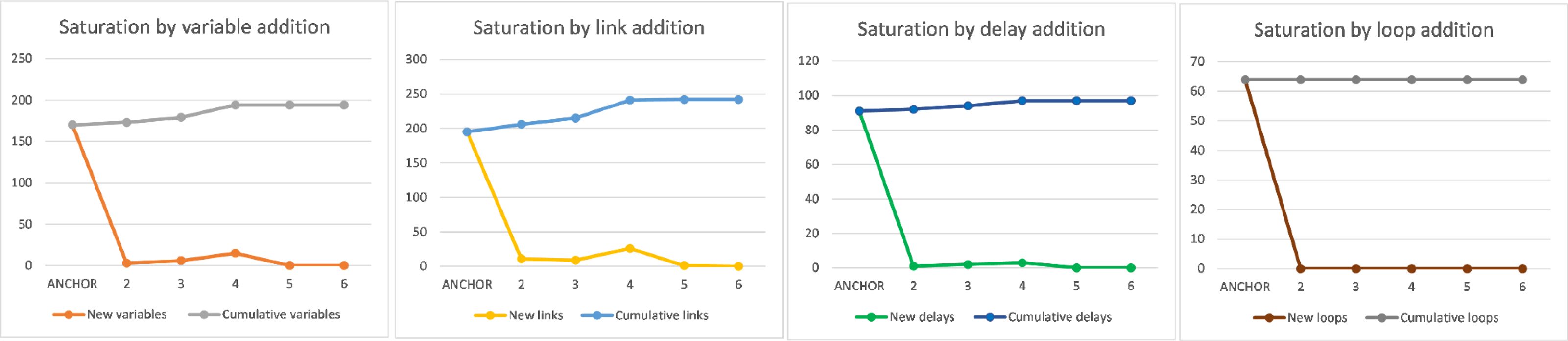

Figure 4. Saturation curves by variable addition, link addition, delay addition, and feedback loop addition, for each additional validation interview added beyond the anchor

In this case the anchor was the aggregated CLD from the initial CLD development step.

Ismail SA, *et al. BMJ Glob Health* 2023; 8:e012399. doi: 10.1136/bmjgh-2023-012399

Appendix 4: Author reflexivity statement

Study conceptualisation	
How does this study address local research and policy priorities?	• The study was designed in partnership with in-country research collaborators in Lebanon (who are named authors on the paper) and prior to initiation of data collection, through scoping discussions with vaccination system stakeholders and with members of the Primary Healthcare Department at the Lebanese MoPH to ensure that it addressed relevant questions;<br>• The CLD was developed entirely through analysis of stakeholder interviews carried out with those working in the vaccination delivery system in Lebanon. It is a visual representation of their "mental models" of how vaccination delivery operates, and variables and causal relationships contributing to trends in vaccination uptake over time, in the context of the shocks described in the study.
How were local researchers involved in study design?	• The core idea for the research was proposed by SAI and developed in consultation with FMF as the main collaborator in Lebanon (SAI and FMF had collaborated on research projects in Lebanon for a number of years previously). Further methodological input was provided by KB and JB as supervisors for the lead author's PhD.
Research management	
How has funding been used to support the local research team(s)?	• Funding was used to pay for local researchers' time on the project.
Data acquisition and analysis	
How are research staff who conducted data collection acknowledged?	• All research staff who contributed to data collection are named authors on the paper (SAI, AN). Research staff supporting the design of the project, data analysis and interpretation are also included as authors (AT-C, FMF, SB, KB, JB).
How have members of the research partnership been provided with access to study data?	• SAI and AN conducted the data collection for the study. The full dataset for the study was held throughout the project by SAI, who performed the analysis.
How were data used to develop analytical skills within the partnership?	• This study was conducted in the context of a PhD for the lead author (SAI). The research supported SAI to demonstrate autonomy and the application of advanced research methods (for academic development purposes). The study enabled AN to build further experience in qualitative interviewing for system dynamics modelling (which she has applied to different research questions previously), in a new subject area.
Data interpretation	
How have research partners collaborated in interpreting study data?	• SAI performed the data analysis, but benefited from extensive input from all co-authors in the interpretation of findings.<br>• AT-C provided core methods advice and input on interpretation from a methodological perspective.<br>• AN and FMF provided essential contextual knowledge.<br>• SB, JB and KB provided overarching methodological, project design and interpretation advice across the project as a whole.
Drafting and revising for intellectual content	
How were research partners supported to	• Through drafting of, and revisions to, this paper and others produced through the PhD project.

Ismail SA, *et al. BMJ Glob Health* 2023; 8:e012399. doi: 10.1136/bmjgh-2023-012399

develop writing skills?	
How will research products be shared to address local needs?	• Results from the research have been shared with the Primary Care Department at the Lebanese MoPH. Research briefs summarising findings across the whole project have been shared with those who participated in the study and a subset of vaccination delivery system stakeholders in Lebanon.<br>• Future plans include the delivery of a webinar to share key findings with relevant stakeholder groups in Lebanon.
Authorship	
How is the leadership, contribution and ownership of this work by LMIC researchers recognised within the authorship?	• The two key in-country collaborators (AN and FMF) at the time this study was conducted have been included as named authors.
How have early career researchers across the partnership been included within the authorship team?	• SAI was a PhD student at the time the study was conducted and this paper is one of the principal outputs from that work. He is included as lead author. AN supported data collection in-country and is listed as a co-author; she is now completing a PhD using related methods in the UK.
How has gender balance been addressed within the authorship?	• The authorship team is balanced – four females (AT-C, AN, SB, JB) and three males (SAI, FMF, KB).
Training	
How has the project contributed to training of LMIC researchers?	• Through the data collection approach, but particularly through analysis and interpretation of data using system dynamics, and through writing of original research papers for submission.
Infrastructure	
How has the project contributed to improvements in local infrastructure?	• Not applicable.
Governance	
What safeguarding procedures were used to protect local study participants and researchers?	• Through ethical approval processes completed prior to the commencement of data collection<br>• Through the anonymisation of data (including quotes) reported in the final study outputs<br>• Through data management and protection procedures throughout the project

BMJ Publishing Group Limited (BMJ) disclaims all liability and responsibility arising from any reliance placed on this supplemental material which has been supplied by the author(s)

Appendix 5: Key system responses identified, by level of action

**Response type** / **System level**	**Absorptive**	**Adaptive**	**Transformative**
Macro	• Focusing infectious disease surveillance activities on COVID-19 **[I]** 🟢	• Initiation of donor response strategy (LCRP and ERP respectively) **[G F]** 🔴🟢🔵<br>• Policy change to reduce cost of vaccination through PHCs **[G F]** 🔴<br>• Policy change to promote access via PHCs if distance to the nearest facility was less than 2.5km **[S]** 🔴<br>• National vaccination campaigns **[S]** 🔴<br>• Introduction of electronic central vaccine stock management system **[M I]** 🔴<br>• Direct payment of MoPH staff salaries by donors **[F]** 🔴	• Policy change to promote nurse-led vaccine delivery via task-shifting **[S W]** 🔴<br>• Policy change to recruit private clinics to the MoPH's network **[S]** 🔴🔵<br>• Introduction of e-record systems **[I]** 🔴
Meso	• Cycling of vaccine stock back to regional (district) level to maintain viability **[M]** 🔵	• Introduction of border crossing vaccination sites **[S]** 🔴<br>• Scale-up of MMU use **[S]** 🔴🔵<br>• Implementing partner subsidies for vaccination delivery **[F]** 🔴🔵<br>• Scale-up of community engagement activities **[P]** 🔴🟢🔵<br>• Introduction of solar fridges **[M]** 🔴<br>• Change in the currency of implementing partner funding to facilities **[F]** 🔵	• Introduction of payment-by-results to support task-shifting **[F]** 🔴

		• Introduction of supplementary payments by implementing partners to improve workforce retention **[F W]** 🔵	
Micro	• Temporary vaccination clinic closures **[S]** 🔴🟢🔵<br>• Changes to clinic working hours **[S]** 🟢🔵<br>• Changes to clinic staffing levels **[W]** 🟢<br>• Reversion to pen-and-paper clinic records (when e-systems not operational) **[I]** 🔵<br>• Improved health worker skills and experience through accumulated clinical exposure **[W]** 🔴	• Staff training (cultural understanding, refugee needs, infection prevention and control and other topics) **[W]** 🔴🟢<br>• Introduction of online and social media-based community engagement activities **[P]** 🟢🔵<br>• Increased recruitment of entry-level healthcare workers and interns to support service delivery **[W]** 🔵	

Table 1. Key system responses identified by the main system level of action, and according to the category of response.

System level of action is given in the table rows; the category of response by the columns. Coloured dots indicate the points at which each response was observed (red dot = refugee crisis response; green dot = COVID-19; blue dot = the economic crisis). Bold letters indicate the kind of intervention implemented, using the WHO health system building blocks (F = financing; G = governance; I = information; M = medicines and technologies; P = people; S = service delivery; W = workforce)

Ismail SA, *et al. BMJ Glob Health* 2023; 8:e012399. doi: 10.1136/bmjgh-2023-012399
